# Mechanistic insights of the left ventricle structure and fibrosis in the arrhythmogenic mitral valve prolapse

**DOI:** 10.21542/gcsp.2018.4

**Published:** 2018-03-14

**Authors:** Leticia Fernández-Friera, Rafael Salguero, Luca Vannini, Ana Fidalgo Argüelles, Fernando Arribas, Jorge Solís

**Affiliations:** 1 Centro Nacional de Investigaciones Cardiovasculares Carlos III (CNIC), Madrid, Spain; 2 HM Hospitales-Centro Integral de Enfermedades Cardiovasculares HM-CIEC, Madrid, Spain; 3 Hospital Universitario 12 de Octubre, Madrid, Spain; 4 Universidad Rey Juan Carlos (PhD Etudent in Epidemiology and Public Health), Madrid

## Abstract

Mitral valve prolapse (MVP) is a common and benign condition. However, some anatomic forms have been recently associated with life-threatening ventricular arrhythmias and sudden cardiac death. Imaging MVP holds the promise of individualized MVP risk assessment. Noninvasive imaging techniques available today are playing an increasingly important role in the diagnosis, prognosis and monitoring of MVP. In this article, we will review the current evidence on arrhythmogenic MVP, with special focus on the utility of echocardiography and CMR for identifying benign and “malignant” forms of MVP. The clinical relevance of this manuscript lies in the value of imaging technology to improve MVP risk prediction, including those arrhythmic-MVP cases with a higher risk of sudden cardiac death.

## Introduction

Mitral valve prolapse (MVP) is a common disorder affecting 2-3% of the general population^1,2^. It is characterized by fibromyxomatous changes in one or both mitral leaflets, which eventually causes a displacement into the left atrium^3,4^. Although MVP is a benign condition, it is the most common cause of isolated mitral regurgitation (MR) that requires surgical repair^5^. Other complications, such as congestive heart failure, endocarditis, stroke and tachyarrhythmias, have been associated with MVP. Furthermore, life-threatening ventricular arrhythmias and sudden cardiac death (SCD) have been recently linked with MVP patients. In fact, there is an important increased risk of atrial and ventricular tachyarrhythmias in MVP patients (50% to 60%) and SCD in these patients (0.4% to 2% per year)^6–8^, even in the absence of heart failure or significant MR.

Sarano et al.^9^ were the first group to describe a subset of MVP patients at high risk of malignant arrhythmias with a specific phenotype, characterized by bileaflet involvement, female sex with mid-systolic click on auscultation, T-wave abnormalities on inferior leads and RBBB-type or polymorphic ventricular arrhythmias, resulting in a “new” MVP entity, called arrhythmogenic MVP.

The normal mitral valve (MV) is a dynamic three-dimensional system, which consists of the mitral annulus, the anterior and posterior mitral leaflets, the chordae tendineae and the papillary muscles (PMs). Its proper function depends on the integrity and harmonious interaction of all these components. MVP entity, including arrhythmogenic MVP, affects all the components of the mitral valve system with histological, macroscopic-anatomical changes and finally, an altered function. Beyond the complex mitral apparatus, several studies have suggested a direct impact of the prolapsing valve on the left ventricular (LV) structure and adjacent myocardium to the MV, potentially causing a secondary cardiomyopathy^3^ . LV damage thus could play an important role in the development of cardiac symptoms in MVP patients, especially in arrhythmogenic MVP where the LV seems to have a pivotal role^9^.

In this context, a detailed study of both the anatomy and function of the entire MV system and the LV myocardium is essential in the diagnosis and prognosis of patients with MVP. Echocardiography is usually the first-line imaging tool for the assessment of MVP patients^10^. Cardiac magnetic resonance (CMR) represents an exhaustive and unique imaging tool for the comprehensive assessment of MVP, with a special utility in arrhythmogenic MVP patients because it provides myocardial tissue characterization, high temporal and spatial resolution images and unlimited spatial planes without acoustic windows limitations.

In this article, we will review the current evidence on arrhythmogenic MVP, with special focus on the utility of echocardiography and CMR for identifying benign and “malignant” forms of MVP. The clinical relevance of this manuscript lies in the value of imaging technology to improve MVP risk prediction, including those arrhythmic-MVP cases with a higher risk of SCD. Noninvasive imaging will help to identify non-benign forms of MVP who are in need of intensive prevention or even, early therapeutic intervention.

## Echocardiography in MVP patients

Transthoracic echocardiography (TTE) is the gold standard for diagnosis and follow-up of MVP^11^ because it is an available, inexpensive and non-invasive technique with high temporal and spatial resolution. Echocardiography allows simultaneous morphological and functional assessment. Two-dimensional (2D TTE) and three-dimensional (3D TTE) echocardiography provides detailed assessment of MV function and morphology, LV size and function, and Doppler echocardiography allows hemodynamic quantification^12,13^.

### Geometry: Mitral valve morphology

MVP by echocardiography is defined by 2D TTE as a single or bileaflet displacement of at least 2 mm beyond the annular plane in parasternal long-axis view, independently of leaflet thickening. Echocardiography allows evaluating leaflet characteristics, especially length and thickness, mitral annulus diameter, and MR severity. Mid-leaflet thickness, which is normally 2–3 mm, is measured usually in parasternal long-axis view in diastole. This measurement depends on the acquisition setting (acoustic wavelength and harmonics)^14^ and is increased in up to 40% of patients with severe MVP. In patients with MVP, leaflets are generally elongated and thickened, and the mitral annulus is enlarged^15^.

MVP is defined as classic when the leaflets thickness is >5 mm and non-classic if there is <5 mm-thickness^3^. Regarding MVP geometry, Sanfilippo et al.^3^ described by 2D echocardiography a PM displacement or mechanical traction toward the mitral annulus due to a combination of leaflet and chordal extensibility, redundancy and elongation. Authors explained that a traction force over leaflet is due to the product of leaflet area and LV pressure and is increased in MVP due to larger leaflet area and annulus. Abnormal mechanical traction of the posterior papillary muscle could be associated with abnormal electrophysiological characteristics in MVP patients^16^.

The use of 3D TTE imaging has shown the real geometry of mitral annulus. Classically, mitral annulus had been considered a planar structure, however, Levine et al.^3^ demonstrated that it has a saddle-shaped with upward concave in the anterior-posterior axis and downward concave in the medium-lateral one. Anterior and posterior mitral leaflets are usually anatomically divided into three segments according to Carpentier’s nomenclature: three posterior scallops, the lateral (P1), middle (P2), and medial (P3) and three anterior scallops, the lateral (A1), middle (A2) and medial (A3)^17,18^.

Mitral annular disjunction (MAD) is defined as a separation between the atrial wall–MV junction and LV attachment. This is a structural abnormality occurring in MVP, related recently to arrhythmic MVP with LV fibrosis in CMR studies^19^. Scarce data exist on the 3D geometry of MAD and its functional implication. Recently described by Lee at al^20^ in a population of MVP by 3D TEE, MAD was present in 42% of patients with MVP, the disjunctive annulus is decoupled functionally from the ventricle, leading to paradoxical annular dynamics with systolic expansion and flattening.

**Figure 1.**
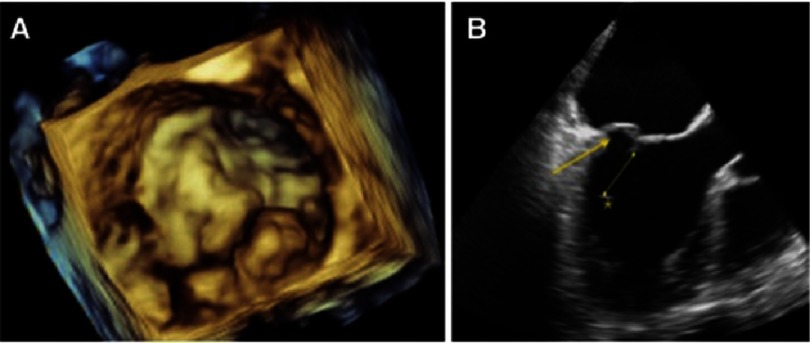
3D transesophageal echocardiography surgical view of P1-P2 segments of a mitral valve prolapse (A). 2D off line reconstruction from 3D full volume LV acquisition. Arrow: P2 prolapse, Line: Primary Chords elongation. Asterisk shows a papillary muscle tip displacement and high intensity echogenicity.

However, TTE may not be accurate to visualize the entire MV anatomy. By taking into account several planes of imaging, 2D transesophageal echocardiography (TEE) is more effective in identifying MV anatomy. 3D TEE has the additional advantage of simulating the surgeon’s view and allows slicing the acquired 3D data in every dimension until the optimal and desired 2D view is obtained^18,21,22^. Also, 3D TEE is superior in identifying the prolapsing segment and defining the MV anatomy over 2D TEE (Figure 1). Moreover, it allows with only one acquisition, quantification of annular dimension, leaflet area, prolapsed volume (volume contained between mitral annulus and the prolapsed leaflet), prolapsed height (distance from the mitral annulus to the prolpased leaflet) and PMs mechanics^21^. All this information is very valuable for surgeons and clinicians.

For example, 3D TEE has been shown to be of value in distinguishing between two different MVP entities^23^: 1) Barlow’s disease, characterized by excess tissue and significantly enlarged leaflets and mitral annulus, and 2) Fibroelastic deficiency, characterized by localized involvement of the leaflet with healthy adjacent segments^17^. This is important because the more extensive involvement of valvular and subvalvular apparatus, the higher the complication rates with poor surgical outcomes.

Thus, prolapsed volume and prolapsed height have recently been identified as possible discriminative factors for the diagnosis of Barlow’s disease and Fibroelastic deficiency^20,23^. In Barlow’s disease (compared to Fibroelastic deficiency), there is an increased prolapse height and prolapse volume^24^, the mitral annulus is larger and present an abnormal enlargement, especially a intercommissural diameter in late-systole and saddle-shape accentuation in early-systole, with a global poor contraction. In severe myxomatous valve disease, the mitral annulus seems to increase flattening which may lead to severe MR and chordal rupture^25^.

### Mitral regurgitation

MR in MVP patients is a consequence of annular enlargement, leaflet redundancy and chordae elongation or rupture, leading to inadequate leaflet coaptation^13,18^. The American Society of Echocardiography and European Association of Echocardiography recommendations for MR assessment highlight the importance of an integrated approach, including semiquantitative and quantitative methods^18,26^. The vena contracta width and proximal isovelocity surface area (PISA) are the preferred methods in patients with MVP. Phasic changes of MR occur in MVP and the effective regurgitant orifice (ERO) increases throughout systole, leading to inaccurate quantification of MR severity. However, properly timed measurements of PISA allow an accurate estimation of the overall ERO and regurgitant volume^24,27^.

### Myocardial tissue characterization: Speckle tracking echocardiography

Although echocardiography does not provide myocardial characterization as well as CMR, the novel myocardial deformation analysis by 2D speckle tracking echocardiography allows the identification of subclinical and specific myocardial deformation abnormalities in MVP. Subclinical LV longitudinal dysfunction has been described since the discovery of MR^20^. MVP patients revealed decreased longitudinal and circumferential peak longitudinal strain and absolute values of strain rate in septal segments^28^. Other studies in different MVP populations did not find decreased longitudinal function, probably related to early stages of LV remodelling, but they detected temporal patterns of myocardial deformation^29^. The authors suggested that the mechanical interaction between MVP and LV wall through chordae and PMs could potentially alter temporal segmental myocardial deformation patterns. Thus, the myocardial segments affected might present electrical vulnerability that could be linked with arrhythmias.

## Cardiac magnetic resonance in MVP patients

CMR is a non-invasive imaging technique that allows a comprehensive characterization of MVP, including assessment of MV morphology, LV structure, MR severity and myocardial tissue characteristics in a single examination. Because this technique provides a more profound look into the biology of the valve and its linked myocardium, it is becoming a valuable tool in complex MVP patients, especially in arrhythmic and genetic familial cases^30,31^, as a complement to the classic echocardiography. CMR also has the ability to acquire unlimited imaging planes and to overcome the limitation of poor acoustic windows, often presented in TTE. It is also less invasive than transesophageal echocardiography. Here, we explore the overall utility of CMR in MVP, with special interest on arrhythmogenic MVP.

### Geometry: Mitral valve and left ventricle morphology

#### Mitral valve morphology

**Figure 2.**
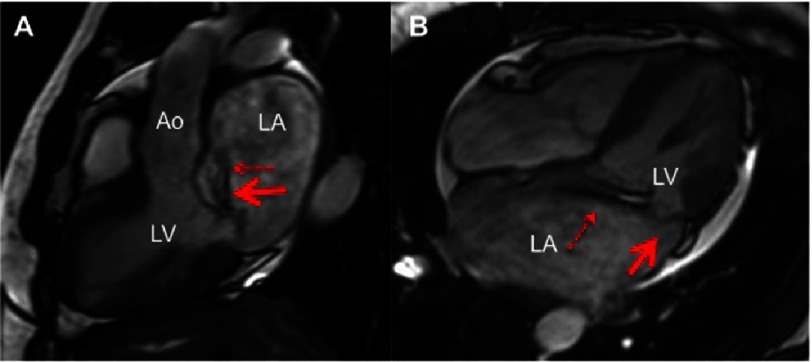
Cine CMR images on left ventricle outflow tract (A) and a 4 chamber view (B) where a posterior mitral valve prolapse is visualized (large arrow) with an eccentric jet of mitral regurgitation (small arrow). Mild pericardial effusion (PE). LV, left ventricle; LA, left atrium; Ao, aorta.

The diagnostic criteria for MVP using CMR are similar to TTE. As compared to the echocardiographic gold standard, MVP on CMR images is defined as a >2 mm mitral leaflet excursion into the left atrium in the LV outflow tract (LVOT) long-axis view, equivalent to a 3-chamber view on echocardiography^20^ (Figure 2). CMR has also the ability to provide a detail MV mapping with a good definition of the prolapsing segments^20,32^. Classic non-contrast cine CMR sequences allow accurate assessment of the MV leaflet and the specific scallop/s affected. This information should be always provided pre-operatively to surgeons because it is crucial to determine the type of surgery and predict the complexity of the surgical repair ahead of time. Other CMR features described in MVP patients include thicker and longer posterior leaflets and larger indexed mitral annular diameters compared to controls. These features showed an excellent correlation compared to echocardiography measurements^20^.

**Figure 3.**
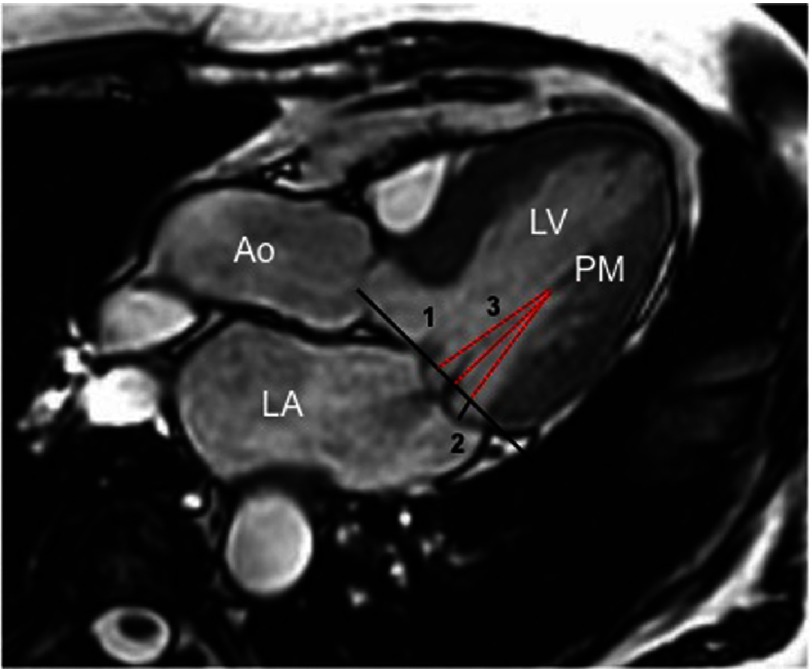
Bileaflet mitral valve prolapse on cine CMR images. 1, Mitral annulus; 2, Prolapsing distance; 3, Distance between the PM and mitral annulus. LV, left ventricle; LA, left atrium; PM, papillary muscle.

From a technical point of view, all these measurements are obtained from a LVOT stack (6 to 8 non-gap 5 mm-slices) across the entire MV, perpendicular to the long axis of the valve using breath-hold, retrospectively electrocardiogram-gated steady state free-precession images. Further MV morphological characteristics have also been described in MVP, including (Figure 3):

- The anterior and posterior displacement, defined as the maximum excursion of the leaflets during systole beyond the mitral annular diameter.
- The distance between the PMs and the mitral leaflet coaptation point and the anterior and posterior leaflets, respectively.
- The anterior and posterior leaflet thickening and length in diastole.
- The mitral valve annulus dimensions, measured at the end-systole in the 2-, 3- and 4-chamber views^10^. Mitral annulus is defined by a line connecting the inferolateral mitral annulus to the aortomitral junction. This measurement might be important when a MV prosthesis implantation or repair is planned.

Above all, anterior leaflet length, posterior leaflet thickness and displacement, and the presence of flail have been shown to be the best determinants of the MR severity in the context of MVP patients^33^. These findings might be important because the prediction of the presence and severity of MR may be valuable for risk stratification, follow-up recommendations and surgical decision-making.

#### LV morphology and function

Interestingly, previous studies have suggested a direct impact of the prolapsing valve on ventricular structure, potentially causing a secondary cardiomyopathy. CMR imaging can provide quantitative determination of ventricular volumes, mass and function by high-resolution images, giving an overview of the LV remodelling. This is particularly useful for follow up of MVP patients with significant MR^34,35^.

LV volumes are calculated by manually traced of LV endocardial contours in end-diastolic and end-systolic phases on steady-state free precession cine images acquired across the entire LV (contiguous 10-15 short axis slices from base to apex). Using the Simpson’s method (LV end-diastolic volume-LV end-systolic volume/LV end-diastolic volume), the LV ejection fraction can be also obtained. LV mass is measured by tracing the LV epicardial contours and the total myocardial volume contained within the endo- and epicontours is multiplied by myocardial density (1.05 g/cm^3^)^36,37^. The extent and distribution of LV hypertrophy in patients with MVP is also important. Zia et al.^38^ showed that MVP is associated with focal basal LV hypertrophy, particularly in the inferolateral and anterolateral segments. Also, they reported that there is good correlation between the excursion of the mitral valve annulus and the degree of relative LV hypertrophy, suggesting that locally increased myocardial function could be responsible for this remodelling.

**Figure 4.**
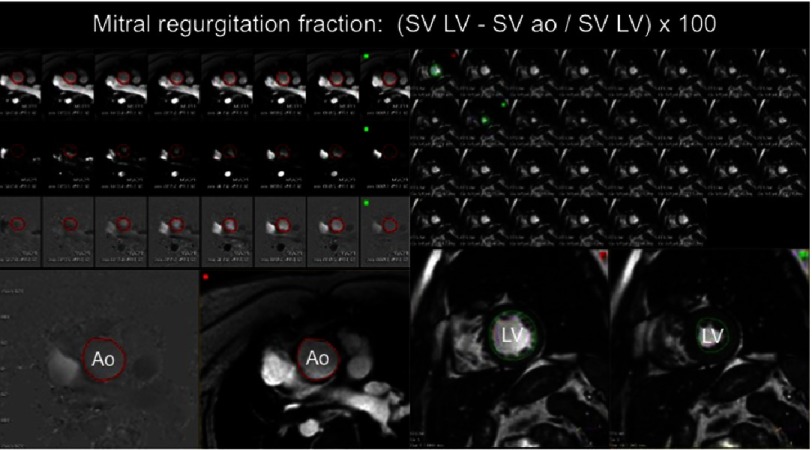
On the left, postprocessing of phase contrast images on the aorta to obtain aortic stroke volume (aortic SV) thougthout the cardiac cycle. On the right, postprocessing of SSFP images to obtain left ventricle stroke volume (LV SV) after manual tracing of end-diastolic and end-systolic contours. Mitral regurgitation fraction is calculated as: (LV SV − aortic SV / LV SV) × 100. Ao: aorta.

**Figure 5.**
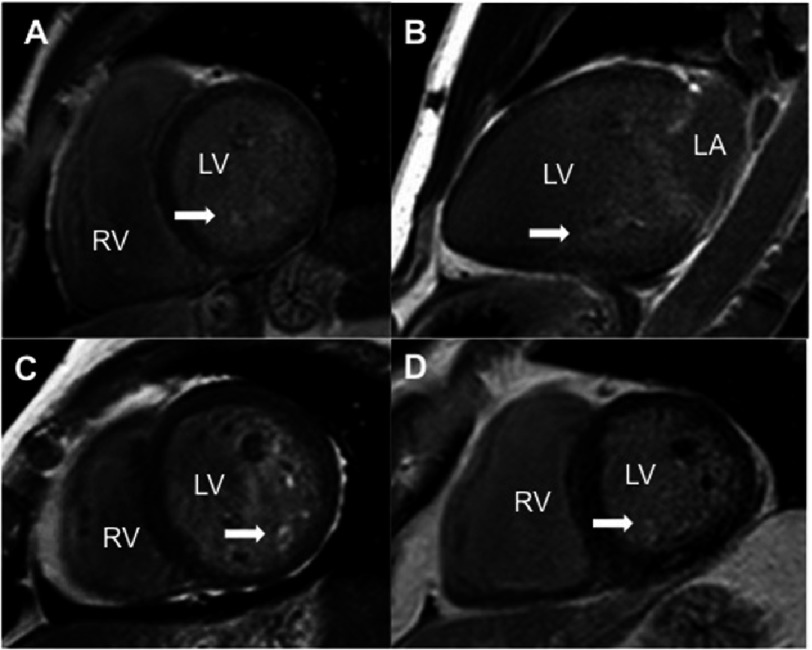
Late gadolinium enhancement CMR images of different MVP patients to show increased uptake (white area) at the papillary muscle level (white arrows). A, C, D, short axis views; B, Two chamber view. LV, left ventricle; LA, left atrium; PM, papillary muscle.

Another CMR application is the evaluation of the PMs function^20^. Functional CMR imaging, such as phase-contrast velocity sequences, can directly measure the motion of the PMs with high temporal and spatial resolution. These techniques assess the excursion and velocity of PMs tips during ventricular systole. Unlike tissue Doppler imaging in echocardiography, no angle dependence is present with phase-contrast CMR methods. Abnormal traction and excursion of PMs can be observed in MVP and can adversely affect the electrophysiologic stability of the underlying myocardium. In fact, there is described an increased peak PMs systolic velocity and maximum PMs excursion in MVP patients compared to controls.

### Mitral regurgitation

Accurate grading of MR severity is crucial for appropriate management. However, the use of echocardiography may be challenging in some complex cases with MVP. Phase-contrast CMR flow imaging has an added value in this scenario because it allows accurate MR quantification. Technically, a perpendicular plane to the ascending aorta at the pulmonary artery bifurcation level is performed using velocity-encoded gradient echo sequences using the minimum upper velocity limit without signal aliasing^36^.

The inner contour of the aorta is traced in each cardiac phase to calculate the aortic stroke volume. Then, the MR volume is calculated as the difference between LV stroke volume (obtained from the cine analysis) and the aortic stroke volume (Figure 4). The MR fraction is obtained by dividing MR volume and LV stroke volume. MR categories are graded as:

0: none to trace (0% to 5%)
1: mild (5% to 16%)
2: moderate (16% to 25%)
3 moderate to severe: (25% to 48%)
4: severe (>48%)^35^.

It has been demonstrated that phase-contrast CMR imaging has low variability and excellent reproducibility^37^, presents the advantage to be independent of the effects of tricuspid and pulmonary regurgitation and allows correction for aortic regurgitation.

### Myocardial tissue characterization: Focal and diffuse fibrosis

#### Focal myocardial fibrosis

A unique feature of CMR is the ability to characterize myocardial or vascular tissues. Contrast agents allow the visualization of macroscopic scars or fibrotic tissue as bright areas into the normal myocardial detected as black using late gadolinium enhancement imaging (Figure 5). Focal fibrosis has provided novel insights into the etiology and risk assessment of different pathologies, mainly cardiomyopathies, and it has been expanded to valvular heart disease. In MVP patients, several studies have reported delayed enhancement in the PMs, probably as a result of continuous chordae tension. For example, Han et al.^20^ demonstrated for the first time that CMR identifies myocardial fibrosis involving the PMs in MVP. Late gadolinium imaging to assess focal fibrosis is acquired in different LV planes 5 to 10 minutes after a bolus of contrast agent injection (Gadolinium 0.2 mmol/kg) using inversion recovery sequences.

#### Diffuse myocardial fibrosis

Another advantage of CMR is the ability of detecting increased extracelullar space by T1 mapping^39^. These increased extracelullar space may represent fibrosis or proteoglycan depositions, as demonstrated in previous studies using myocardial histological analysis. Recently, it has been described a relationship between chronic volume overload in asymptomatic chronic primary degenerative MR and myocardial fibrosis using T1 mapping techniques^40^. Moreover, the myocardial fibrosis was associated with reduced myocardial deformation and reduced exercise capacity. Thus, T1 mapping, by detecting increased extracelullar volume as a surrogate of diffuse fibrosis, could be considered as another valuable marker to decide the optimum surgery timing in asymptomatic patients with chronic severe primary degenerative MR.

**Figure 6.**
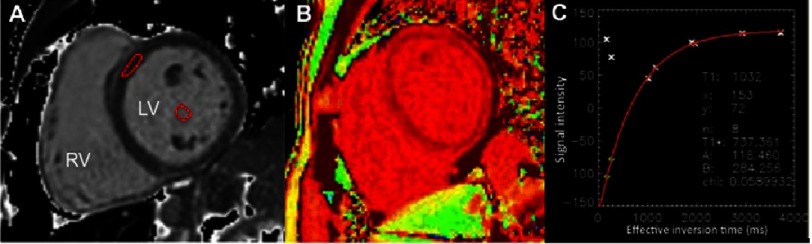
CMR T1 mapping imaging of the left ventricle (LV) where a region of interest (red ROI) has been placed in the septal wall and in the blood pool to calculate extracellular volume (A). T1 mapping in a color map (B) and T1 mapping graphs (C).

Briefly, CMR T1 mapping may be assessed with different sequences^41,42^. The most widely used is the MOLLI sequence (ECG-gated single shot modified Lock Locker inversion recovery)^43^. Using MOLLI sequences, T1 maps are acquired before and after a contrast infusion or bolus injection to compare myocardial relaxation times changes^44^. For the analysis, a region of interest is usually manually traced in the septum wall (Figure 6). The signal intensity vs. time curve for each segment and blood pool will be used to determine a segmental T1 through exponential fitting and its reciprocal, R1. The slope of the linear relationship between R1 for myocardium vs. blood from all R1 measurements (before and after gadolinium administration) defines the partition coefficient for gadolinium. To obtain the myocardial volume of distribution of gadolinium, or extracellular volume fraction, the partition coefficient will be multiplied by (1- hematocrit/100) to obtain the “fibrosis index”^42^.

## CMR in patients with arrhythmogenic MVP

The increased risk of life-threatening ventricular arrhythmias and SCD in a subgroup of MVP patients suggests an underlying myocardial involvement in the pathophysiology mechanisms of electric instability. Detecting imaging markers to identify high-risk candidates to suffer malignant arrhythmia has thus important clinical implications. MVP is a relatively common echocardiographic finding but only a small proportion is linked to ventricular arrhythmias. CMR imaging, besides the classic MVP characterization described above, can help in the identification of arrhythmic MVP patients by assessing mainly tissue composition and morphological and functional MV characteristics.

### Myocardial tissue characterization: Focal and diffuse fibrosis

In 2015, new imaging features of arrhythmic patients with MVP were described to provide additional information over the classic prognostic markers^9^. Basso et al.^45^ identified for the first time focal myocardial scarring at the inferobasal LV wall under the posterior leaflet in alive MVP patients with threatening arrhythmias. This scar, detected by CMR, could be the origin of the RBBB-type ventricular arrhythmias, even in the absence of significant MR. Authors also described fibrosis as patchy and interspersed within surviving hypertrophic cardiomyocytes in sudden death patients. Previously, the presence of fibrosis in MVP has been described only at the PM^20^ but in patients with moderate to severe MR.

MVP-fibrosis formation could be explained by different causes. The most probable hypothesis is a myocardial stretch by the prolapsing leaflets and elongated chordae that could act as a trigger of electric instability. In line with previous reports, Basso et al.^45^ proposed that MVP could be considered a cardiomyopathy with significant myocardial involvement^46–48^ because of the MVP itself. LV abnormalities, including regional hypercontractility with myocyte hypertrophy, acts as the cause of MV geometry disruption with abnormal tension on the chordae and leaflets leading eventually to fibrous tissue repair. Interestingly, the amount of scar in MVP is relatively small, in contrast to other pathologies were larger scar burden are associated with worse prognosis.

Diffuse rather than focal fibrosis have also been associated with MVP^40^. In the context of arrhythmogenic MVP, Delling et al.^49^ evaluated T1 mapping techniques by CMR to assess extracelullar volume as a surrogate of diffuse fibrosis. The authors found shorter post-contrast T1 times in patients with MVP and arrhythmias compared with MVP patients without arrhythmias, even in the absence of focal fibrosis. Thus, diffuse myocardial fibrosis may be substrate of ventricular arrhythmia as a consequence of LV remodelling secondary to MR.

The authors suggested that diffuse fibrosis could be the consequence of profibrotic cytokine high levels. These hypotheses are based on previous animal and *in vitro* studies that have demonstrated that overexpression of transforming growth factor (TGF)-β contributes to fibrosis and matrix remodelling in MVP^50,51^. Furthermore, expression of TGF-β was significantly increased in patients with SCD compared with controls, suggesting the role of TGF-β as a potential mediator of interstitial remodelling in sudden death^52^. Consequently, other mechanisms of arrhythmias may be involved besides focal scar, such as diffuse fibrosis, as arrhythmias may also been present in the absence of focal fibrosis. In conclusion, T1 mapping may become an additional marker of arrhythmic risk in MVP as a predictor of SCD.

### Geometry: Morphological and functional MV characteristics

**Figure 7.**
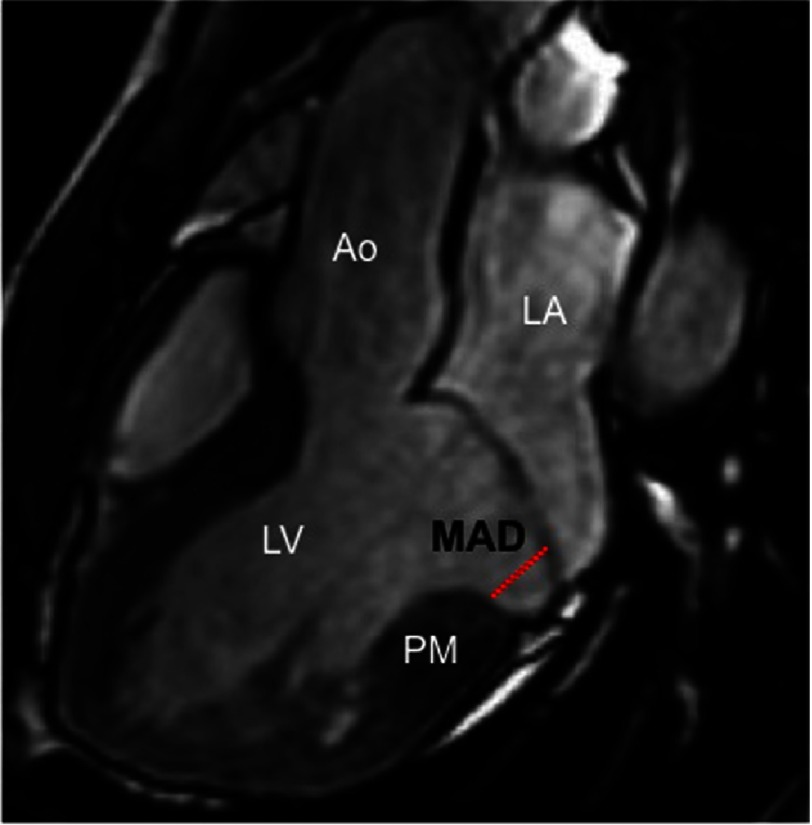
Bileaflet mitral valve prolapse on cine CMR images in a patient with arrhythmogenic mitral valve prolapse. This figure shows an enlarged mitral annulus disjunction (MAD). LV, left ventricle; LA, left atrium; PM, papillary muscle.

Classic pathology studies in patients with MVP who died suddenly focused mostly on MV structural alterations, leaflet length and thickness, and the presence and extent of endocardial plaques^53,54^. More recently, several morphofunctional abnormalities of the MV that could explain a regional mechanical myocardial stretch have been described, including^19^ (Figure 7): 1) Mitral annulus disjunction (MAD), defined as a the separation between the left atrium valve junction and the top of the LV inferobasal wall during end systole; 2) End-systolic and end-diastolic mitral annular dimensions; 3) Posterior systolic curling, measured by tracing a line between the top of LV inferobasal wall and the LA wall–posterior MV leaflet junction, and from this line, a perpendicular line to the lower limit of the mitral annulus during end systole; 4) Basal to mid LV wall thickness ratio.

Marra et al.^19^ found that all these parameters were higher in arrhythmic MVP patients with focal scar compared to MVP cases without scar using CMR. In addition, histology of the mitral annulus showed longer MAD in sudden death patients with MVP than in patients without MVP. Indeed, the higher the magnitude of MAD, the higher the probability of having ventricular arrhythmias. Authors concluded that MAD is a constant feature of arrhythmic MVP with LV fibrosis. This concept of MAD was first introduced by Hutchins et al.^55^ in disease pathogenesis in patients with severe MR. The excessive mobility of the leaflets, caused by myxomatous degeneration, posterior systolic curling and MAD, accounts for a systolic mechanical stretch of the myocardial linked to the MV (inferobasal wall and papillary muscles), eventually leading to myocardial hypertrophy and scarring. These mitral annulus abnormalities may identify MVP patients who would need arrhythmic risk stratification. Interestingly, a paradoxical systolic increase of the mitral annulus diameter was also observed in this study causing an alteration of the geometry and function of the mitral ring.

The role of abnormal mechanical forces in the formation of ventricular arrhythmias was indirectly demonstrated by Vaidya et al.^56^ They showed in a series of cases that the surgical correction of bileaflet MVP was associated with a reduction in malignant arrhythmias. MV repair could thus relieve the mechanical stretch of the myocardium, thus leading to a reduction of arrhythmias. All these findings by CMR and MVP studies provide a deep and better understanding of the pathophysiology of ventricular arrhythmias in MVP.

## Pathophysiology of ventricular arrhythmias in MVP

Beyond extracardiac causes as autonomic nervous dysfunction, the mechanism of arrhythmogenic MVP may be related to abnormalities in the MV system and the involved myocardium. As mentioned above, bileaflet involvement, larger MAD or severe MV geometry distortion may represent a subset of patients and risk markers to develop malignant ventricular arrhythmias^19^. Different mechanisms of ventricular arrhythmias may be involved in MVP. Single ventricular premature beats (VPB) from the papillary muscle (RBB mostly with superior axis) may represent focal activity elicited by mechanical actions on this sensible area for arrhythmias^57,58^. In fact PMs sustained arrhythmias have been recognized as a different entity from fascicular tachycardia^59,60^.

The infrequent malignant MVP, as compared to the MVP population with VPB, probably underlines the role of a substrate for sustained ventricular arrhythmias. Local fibrosis at the PMs, or more extensive fibrosis engaging inferoposterior wall, may well be the substrate. A patchier pattern of fibrosis, compared to that solid one of ischemic myocardial infarction, makes more feasible sustained rapid circuits, resulting more easily in ventricular fibrillation^45^. Although a hidden cardiomyopathy in malignant MVP cannot be excluded, fibrosis might be a more advanced phase in more severe cases because of myocardial remodelling. Inducibility of sustained ventricular in MVP patients with VPB and symptoms of sustained ventricular arrhythmias and prior sustained ventricular tachyarrhythmias or ventricular fibrillation may support the difference of focal triggers versus substrate for reentry^61^.

Identification of these high-risk patients is important in order to consider different treatments. Ablation of the trigger could be feasible since it has been successfully reported in other patients with ventricular fibrillation^62^. Antiarrhythmic effect of surgical reparation in early phases is still to be demonstrated since surgery in patients with sustained ventricular arrhythmia failed to avoid inducibility^7^. For other patients, like those with extensive scarring, frequent complex multifocal VPB or inducible sustained ventricular arrhythmias, symptoms of brain hypoperfusion, Implantable Cardioverter Defibrillator (ICD) implantation could be considered if the risk is evaluated to be high enough.

## Conclusions

Although MVP is a benign condition, there is a subset of patients at high risk of life-threatening ventricular arrhythmias and SCD with a specific phenotype, resulting in a “new” MVP entity, called arrhythmogenic MVP. Malignant arrhythmias in MVP may be generated due to the combination of abnormal LV myocardial substrate (myocardial fibrosis) and a continuous trigger (mechanical stretch). Noninvasive imaging technology, especially CMR, may help to identify non-benign forms of MVP who are in need of intensive prevention or even, early therapeutic intervention. The presence of focal fibrosis in the basal inferolateral LV wall, probably the area more related to the arrhythmias’ origin, diffuse LV fibrosis and morphological MV characteristics, such as increased MAD, diffuse leaflet thickening, elongation and redundancy of chordae, have been linked to arrhythmogenic MVP. Thus, a proper assessment of MVP for clinical decision-making and prognosis, should not be only limited to the MV, but rather include a comprehensive evaluation of the myocardial tissue composition and LV structure, as a potential cardiomyopathy.
